# Effect of SSRIs on Resting-State Functional Brain Networks in Adolescents with Major Depressive Disorder

**DOI:** 10.3390/jcm10194322

**Published:** 2021-09-23

**Authors:** Shu-Hsien Chu, Keshab K. Parhi, Melinda Westlund Schreiner, Christophe Lenglet, Bryon A. Mueller, Bonnie Klimes-Dougan, Kathryn R. Cullen

**Affiliations:** 1 Department of Electrical & Computer Engineering, University of Minnesota, Minneapolis, MN 55455, USA; chuxx214@umn.edu (S.-H.C.); parhi@umn.edu (K.K.P.); clenglet@umn.edu (C.L.); 2 Department of Psychiatry, Huntsman Mental Health Institute, University of Utah, Salt Lake City, UT 84108, USA; mindy.westlund.schreiner@utah.edu; 3 Department of Radiology, University of Minnesota, Minneapolis, MN 55455, USA; 4 Department of Psychiatry and Behavioral Sciences, University of Minnesota, Minneapolis, MN 55454, USA; muell093@umn.edu; 5 Department of Psychology, University of Minnesota, Minneapolis, MN 55455, USA; klimes@umn.edu

**Keywords:** major depressive disorder (MDD), functional magnetic resonance imaging (fMRI), resting-state functional connectivity (RSFC), frequency-dependent connectivity, network topology

## Abstract

Investigation of brain changes in functional connectivity and functional network topology from receiving 8-week selective serotonin reuptake inhibitor (SSRI) treatments is conducted in 12 unmedicated adolescents with major depressive disorder (MDD) by using wavelet-filtered resting-state functional magnetic resonance imaging (fMRI). Changes are observed in frontal-limbic, temporal, and default mode networks. In particular, topological analysis shows, at the global scale and in the 0.12–0.25 Hz band, that the normalized clustering coefficient and smallworldness of brain networks decreased after treatment. Regional changes in clustering coefficient and efficiency were observed in the bilateral caudal middle frontal gyrus, rostral middle frontal gyrus, superior temporal gyrus, left pars triangularis, putamen, and right superior frontal gyrus. Furthermore, changes of nodal centrality and changes of connectivity associated with these frontal and temporal regions confirm the global topological alternations. Moreover, frequency dependence is observed from FDR-controlled subnetworks for the limbic-cortical connectivity change. In the high-frequency band, the altered connections involve mostly frontal regions, while the altered connections in the low-frequency bands spread to parietal and temporal areas. Due to the limitation of small sample sizes and lack of placebo control, these preliminary findings require confirmation with future work using larger samples. Confirmation of biomarkers associated with treatment could suggest potential avenues for clinical applications such as tracking treatment response and neurobiologically informed treatment optimization.

## 1. Introduction

Major depressive disorder (MDD) is a highly debilitating condition that can result in tragic outcomes such as chronic disability and suicide. The onset of depression is frequent during the adolescent period [1], a time notable for significant brain development and physical and social changes [2,3]. According to the World Health Organization (WHO), more than 10% of adolescents are affected by MDD in the US [4,5]. Moreover, early onset MDD has been shown to increase the risk of developing adult depression [6]. However, current evidence-based treatments including antidepressant medication and cognitive behavioral therapy are only successful in reducing depression in about half to two-thirds of cases [7]. To design and optimize new and more effective treatments, a deeper understanding of the neurobiological basis of existing treatments is needed.

During the last decade, MRI has been extensively applied in studying neural underpinnings of depression. Using structural MRI, previous studies have reported that patients with depression show reduced volumes compared to healthy controls in the anterior cingulate cortex, orbitofrontal cortex, and hippocampus [8,9,10]. Based on diffusion MRI, previous studies have shown disrupted white matter integrity in the limbic system, dorsolateral prefrontal cortex, thalamic fibers, and corpus callosum [11,12,13,14,15]. Additionally, numerous reports have shown disruptions of functional connectivity in the limbic system using functional MRI (fMRI) [16,17,18,19,20]. Taken together, these findings suggest that MDD involves a complex set of connectivity deficits in the fronto-limbic system as well as in other brain networks.

Since MDD involves neural network abnormalities, a growing body of research has been dedicated to understanding how such networks may change after antidepressant treatments [21,22,23,24,25,26]. However, prior studies have largely focused on specific regions of interest, i.e., regional functional activation and functional connections from them, such as the amygdala. For example, in a prior report from the same dataset, as reported here, we investigated treatment-related changes in resting-state functional connectivity between the amygdala and all other voxels in the brain [21,22]. However, it is possible to examine connectivity changes at a more global level in order to assess change in connections among all brain regions. Furthermore, beyond functional connectivity analyses, a more advanced approach to understanding brain networks is to characterize the topological characteristics of the network. Brain network studies have recently shown the promising power of graph-theory based analysis in examining topological properties for brain networks considering cortical/subcortical regions as nodes and anatomical or functional connectivity among regions as edges [27,28,29,30,31]. Using graphical analysis, the *small-world* property has been discovered in both structural and functional networks for healthy brains [32,33,34,35,36]. A small-world network has the property of a high clustering coefficient and a low characteristic path length. These features are associated with efficient local information delivery, i.e., functional segregation, and effective collaboration for distributed information processing, i.e., functional integration, respectively, of the brain network structure [27]. Graph theory analysis has been extensively used to uncover brain network abnormalities in patients with schizophrenia [37,38,39,40], Alzheimer’s disease [41,42,43,44], and depression [14,45,46,47]. These promising results motivate the exploration of treatment-related topological patterns in this work from the rapidly changing adolescent brain networks, which has not been investigated in previous studies. Given the complexity of MDD symptoms and response to treatment, knowledge about possible topographical disruptions in functional brain networks could provide greater direction in treatment selection and development.

**Figure 1:**
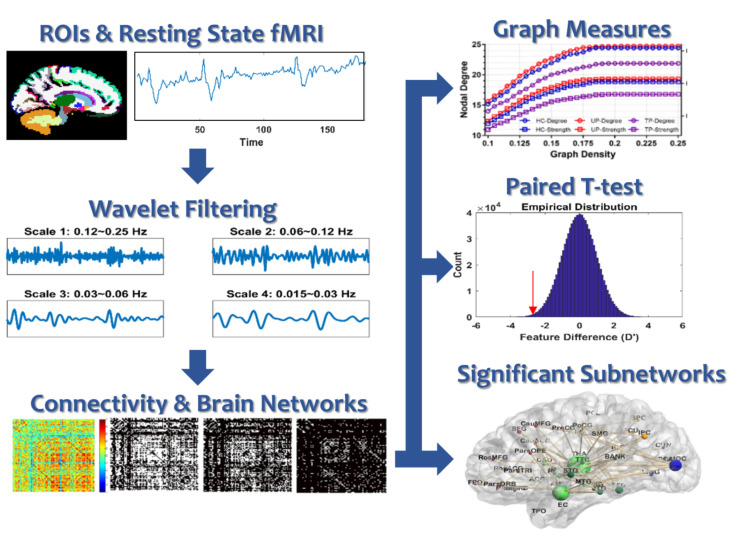
Framework for data analysis.

This study aims to discover treatment-related changes in brain functional network topology and whole-brain connectivity for 12 unmedicated adolescents with MDD before and after receiving an 8-week selective serotonin reuptake inhibitor (SSRI) medication treatment. We specifically analyzed brain networks with frequency-selective resting-state functional connectivity (RSFC) by using filtered fMRI signals in four frequency bands. First, we examine treatment-related changes in a set of topological measurements. Second, we examine treatment-related changes in functional connectivity between pairs of all brain regions. Third, we examine treatment-related change in subnetworks that are composed of the significantly altered connections. Finally, we examine correlations between clinical assessment and brain network features (topological measures and connectivity). The framework for data analysis is shown in Figure 1.

## 2. Material and Methods

### 2.1. Participants

Participants of this study consisting of twelve adolescents aged 12 to 19 years diagnosed primarily with MDD were unmedicated before the baseline scan. They were invited to return for a second neuroimaging scan after they completed 8 weeks of antidepressant treatment under the care of their own provider. They represent a subset of a larger, cross-sectional neuroimaging study [19]. After the informed consent process, all participants completed a comprehensive diagnostic assessment. Interviews were conducted separately with adolescents and parents, and included *Kiddie Schedule for Affective Disorders and Schizophrenia-Present and Lifetime Version* [48] and the *Children’s Depression Rating Scales-Revised* (CDRS-R) [49]. Self-report measures assessing symptoms in the past two weeks included the Beck Depression Inventory II (BDI-II) [50,51] and the Inventory of Depression and Anxiety Symptoms (IDAS) [52,53,54]. The IDAS provides a score for the following symptom dimensions: general depression, dysphoria, lassitude, insomnia, suicidality, appetite loss, appetite gain, ill temper, well-being, social anxiety, panic, and traumatic intrusion. Patients with any of the following exclusionary conditions were excluded: current use of psychotropic medications, intellectual disability, pervasive developmental disorder, substance use disorder, bipolar disorder, and schizophrenia. The demographic information is summarized in Table 1. The experimental procedures involving human subjects described in this paper were approved by the University of Minnesota Institutional Review Board in 2008 (case number 0804S30542).

### 2.2. Pre- and Post-Treatment Clinical Assessment

Diagnosis is based on the *Kiddie Schedule for Affective Disorders and Schizophrenia-Present and Lifetime version* (K-SADS-PL) [48] interviews completed separately with adolescents and parents [21,22,55] and the Beck Depression Inventory-II (BDI-II) [50]. The BDI-II was also administered on the day of the post-treatment scan. Change in depression symptoms was calculated by subtracting the pre-treatment scores from the post-treatment BDI-II scores (negative scores represent improvement as indexed by a drop in depressive symptoms).

### 2.3. Image Acquisition and Processing

MRI scans are conducted by using a Siemens 3 Tesla TIM Trio scanner located at the Center for Magnetic Resonance Research, University of Minnesota. A T1-weighted high-resolution magnetization prepared gradient echo sequence scan is obtained with following parameters: repetition time =2530 ms, echo time =3.65 ms, inversion time =1100 ms, flip angle =7 degrees, 1 mm slices, field of view =256 mm, voxel size 1×1×1 mm ${}^{3}$, and GRAPPA =2.5 min. For the resting-state fMRI (rsfMRI), functional data were acquired by using an echo planar imaging sequence (EPI) with the following: 180 T2–weighted whole-brain functional volumes with 34 interleaved slices of 4 mm thickness and no skip, tilted to AC-PC alignment, repetition time =2000 ms, echo time =30 ms, flip angle = 90 degrees, field of view =220 mm, and 64×64 matrix (voxel size 3.4375×3.4375×4 mm${}^{3}$). Participants are asked to close their eyes and stay awake. Physiological data (respiration and cardiac traces) are collected during the entire scan.

Brain parcellation is performed using Freesurfer (http://surfer.nmr.mgh.harvard.edu, accessed 20 August 2018) following the Desikan–Killiany atlas to create 76 cortical/subcortical regions listed in Table 2 [56]. FreeSurfer output was visually inspected; identified errors were manually corrected on a slice-by-slice basis. The parcellation is registered to the fMRI data using *FLIRT* and *FNIRT* tools in FSL (Functional Magnetic Resonance Imaging of the Brain Software Library; http://www.fmrib.ox.ac.uk/fsl, accessed 20 August 2018) [57,58,59]. Functional images are preprocessed for motion correction, brain extraction, RETROICOR processing (regression of physiological signal, i.e., heart beat and respiration, which were recorded during the scan [60]), geometric distortion correction using the field map, and removing regression of signal from CSF and white matter. Data scrubbing was performed following the method of Power and colleagues [61], excluding any volume with a value for the temporal derivative of time courses’ root mean squared head motion variance exceeding 8 and/or a framewise-dependent value exceeding 0.5, along with the previous volume and the 2 following volumes.

### 2.4. Resting-State Functional Connectivity and Network Construction

Previous studies have found that the functional connectivity between brain regions is frequency dependent [32], and disease-related alterations in functional connectivity are prone to specific frequency bands [44,62,63]. Therefore, in this study, the fMRI signal is filtered using a 4-level stationary discrete wavelet transform (SDWT) [64,65] with `db4’ wavelet into different frequency bands. Unlike traditional decimated wavelet transform, the SDWT satisfies translation-invariance. In SDWT, the downsamplers and upsamplers are removed, and the filter coefficients are upsampled. The wavelet scales approximately correspond to frequency ranges of 0.12–0.25 Hz, 0.06–0.12 Hz, 0.03–0.06 Hz, and 0.015–0.03 Hz, respectively. After SDWT filtering, functional connectivity between brain regions is computed based on the Pearson correlation between the average fMRI time courses at each frequency band separately for each subject. A frequency-specific 76-by-76 undirected graph is constructed based on the 2850 correlation coefficients for each frequency band.

### 2.5. Graph-Theoretic Analysis

Adaptive thresholding is employed to create a series of brain networks corresponding to graph densities from 10% to 50% for network topology analysis. Individual bias is eliminated, and the dynamic range of connectivity is normalized by adaptive thresholding. The core network has the smallest density and contains the top 10% strongest connections; by contrast, the most robust network has the largest density and contains the top 50% strongest connections. The range of densities is chosen to ensure the capability of the small-world measure [66].

#### 2.5.1. Graph Measures

Graph structures are quantified by using graph-based measures including clustering coefficient, characteristic path length, smallworldness, global/local efficiency, participation coefficient, within-module-degree z-score, degree, strength, and betweenness centrality. A complete review of topological brain network measures can be found in the literature [29,67]. Symbols are defined as the following:

- G=(N,E): an undirected graph where *N* is the set of nodes and *E* is the collection of existing connections/links;
- l=(i,j)∈E: the connection between node *i* and *j*;
- $a_{ij}$: the existence of a connection l=(i,j). $a_{ij}=1$ if the link is connected, otherwise $a_{ij}=0$;
- $w_{ij}$: the functional connectivity of a connection l=(i,j);
- $M=\{m|⋃m=Nandm⋂m^{\prime }=\emptyset ,\;\forall m\neq m^{\prime }\}$: the collection of modules of the graph where *m* is a module;
- $m_{i}$: the module that node *i* belongs to.

##### Clustering Coefficient

The clustering coefficient, $C_{i}$, is a measure of the degree to which nodes in a neighborhood tend to cluster together, and it is defined as the ratio of the number of closed triangles to the number of connected triples of vertices in the two-hop neighborhood of a node, *i*. The higher the clustering coefficient is, the more tightly the node is connected to its neighbors. Therefore, a high local clustering coefficient potentially implies high local efficiency.

##### Characteristic Path Length

The characteristic path length $L_{net}$ quantifies the integration ability of a network. The definition of $L_{net}$ is the average distance between any two nodes in the network. To avoid the disconnection problem, i.e., the distance between some nodes is infinity, the harmonic mean version of the original definition is used in this study:

$$L_{net}=\frac{n(n-1)}{\sum_{i\neq j\in N}d_{ij}^{-1}}$$

where *n* is the number of nodes in *N* and $d_{ij}$ is the shortest path length between node *i* and node *j*.

##### Smallworldness

The small-world properties of a network are quantified by the clustering coefficient and the characteristic path length of the network. The clustering coefficient of the entire network $C_{net}$ is the mean $C_{i}$ of all nodes in the network. This global measure quantifies the cliquishness of a network. In order to diagnose the small-world properties, global clustering coefficients and characteristic path lengths are normalized by the same metrics estimated from random networks with the same number of nodes, edges, and degree distribution. The normalized network clustering coefficient is defined as $C_{norm}=C_{net}/C_{rand}$, the normalized characteristic path length is defined as $L_{norm}=L_{net}/L_{rand}$, and the smallworldness of the network is defined as $C_{norm}/L_{norm}$. A small-world network is expected to have high local clustering and low mean path length; therefore, the smallworldness is greater than 1 for a small-world network.

##### Degree

Degree, defined as $k_{i}=\sum_{j\in N}a_{ij}$, measures how well a node is connected to the other nodes, which can also be interpreted as the (nodal) centrality of the node. The degree is defined as the number of binary connections to the node. Therefore, a node with a large nodal degree might be considered a hub.

##### Local Efficiency

Local efficiency, $E_{i}$, is a measure of how efficiently nodes exchange information in the one-hop neighborhood of node *i*, and it is positively related to the clustering coefficient.

##### Modules

A module, *M*, also known as a cluster, is defined as a non-overlapping division of a network that represents the structural segregation in the brain. In a cluster, nodes are abundantly connected, and nodes in different clusters are connected through a few hub regions (nodes). The interpretation of the module formation is that different structural modules are segregated by biological and functional characteristics.

##### Participation Coefficient

Participation coefficient, $y_{i}=1-\sum_{m\in M}{\left(\frac{k_{i}\left(m\right)}{k_{i}}\right)}^{2}$ where $k_{i}\left(m\right)=\sum_{j\in m}a_{ij}$ , assesses the diversity of inter-modular interconnections of individual nodes. The parameter $k_{i}\left(m\right)$ denotes the within-module degree of node *i*. In the brain network, a provincial hub, which is an important part in the facilitation of modular segregation, will have a high within-module degree but a low participation coefficient. Furthermore, nodes having both high within-module degree and participation coefficient facilitate global inter-modular integration as connector hubs.

##### Betweenness Centrality

The betweenness centrality is a measure of centrality defined by the fraction of all shortest paths in the network that contain a given node. Nodes with high values of betweenness centrality participate in a large number of shortest paths.

#### 2.5.2. Graph Measure AUCs

For binary graph measures, we considered the area under curve (AUC) of the measure across a range of graph densities. When the range of graph densities is uniformly sampled, the AUC is just the average measure across graph densities.

### 2.6. Statistical Analyses

In order to assess the significance of a change, *D*, in brain network measures from baseline to post-treatment, we first estimated the empirical distribution of the null hypothesis, $H_{0}$: no significant change due to the treatment, by performing a random sign permutation test. During the test, the feature differences between before and after treatment are randomly assigned with a positive or negative sign to calculate the average difference, $D^{\prime }$, for a realization of the random process. To test the hypothesis, 1,000,000 realizations are implemented. The probability that the average difference, $D^{\prime }$, is greater or smaller (depending on the sign of *D*) than the original measured average difference, *D*, is the empirical tail probability, i.e., *p*-value. With *N* realizations, the best achievable resolution of the *p*-value is $\frac{1}{N}$, which is $10^{-6}$ here. In other words, p-value differences smaller then $10^{-6}$ are not distinguishable, i.e., estimation may be zero for any *p*-value less than $10^{-6}$. Although, the best resolution for random sign permutation tests is set to $10^{-6}$, the resolution is still limited by the sample size. In this study, 12 subjects yield $2^{12}$ combinations and result in the best *p*-value resolution of $2^{-12}=0.0002441$.

As an exploratory study, uncorrected *p*-values are reported, and minimal multiple-comparison correction (corrected for direct comparisons, i.e., by number of regions for region-related metrics and by number of connections for connection-related metrics) is performed with Bonferroni correction to highlight potential changes for 5% false-discovery rate.

In addition to the *p*-value, effect size and power are also reported. The effect size of the treatment-related change was measured using Cohen’s d with pooled standard deviation [68]. The statistical power of the test with significance level 0.05 is calculated based on the mean of changes, the standard deviation of changes, and sample size (N=12), and it is tested against the null hypothesis ($\mu_{0}=0$) [69,70].

In order to assess possible correlations between neural network changes and clinical changes, we conduct regression analysis using *`fitlm’* (Matlab) by taking the changes in brain network measures as main regressors and change in total BDI-II scores as the outcome variable. Additionally, to discover whether baseline network measures might serve as predictors of treatment response, we also perform regression analysis using before-treatment brain network measures as main regressors, again with the change in total BDI-II scores as the outcome variable.

Previous research suggests that analyzing a subnetwork can effectively reduce the number of hypothesis tests for controlling the false positive rate and increase statistical power in connectomic analysis [71,72]. Here, we create a subnetwork consisting of significantly changed connections selected with a false discovery rate (FDR) at level α=5% using Benjamini–Hochberg procedure [73] for each frequency band. In order to examine the broadness of treatment effect to a region in communicating with the other regions, within-subnetwork nodal degree is calculated, which is defined by the number of connections connected to the node in the subnetwork.

## 3. Results

### 3.1. Treatment-Related Changes in Network Topology

#### 3.1.1. Global Topological Metrics

**Figure 2:**
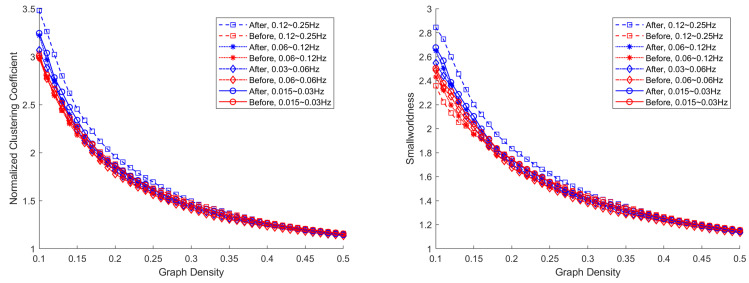
Mean global network measures, normalized clustering coefficient, and smallworldness across graph density in 4 frequency bands for before and after 8-week SSRI treatment.

Figure 2 shows normalized clustering coefficient and smallworldness in mean curves across patients as functions of network densities for baseline and after treatment in all four frequency bands. Curves show results similar to previous studies in the small-world topology that the normalized clustering coefficient is greater than one and approaches one when the network density increases and the smallworldness decreases when the network density increases [32,33,35,38,42]. However, in addition to previous findings, results indicate the clustering coefficient is increased after treatment in all frequency bands for small network densities, i.e., core networks. Given no change in characteristic path length, smallworldness shows consistent change with the clustering coefficient. Regular networks have large clustering coefficient and low smallworldness, and random networks have low clustering coefficient and low smallworldness. The increased clustering coefficient and smallworldness indicate that core network topology has changed toward a small world network that is commonly observed from human brain networks. In terms of statistical significance, the AUC of the normalized clustering coefficient differences has *p*-value ranging from 0.083 to 0.47, and the smallest value is achieved in the 0.12–0.25 Hz band with an effect size of 0.57. For the smallworldness, the *p*-value ranges from 0.143 to 0.431, and the smallest value is achieved in the 0.12–0.25 Hz band with an effect size of 0.4765.

#### 3.1.2. Local Topological Metrics

**Figure 3:**
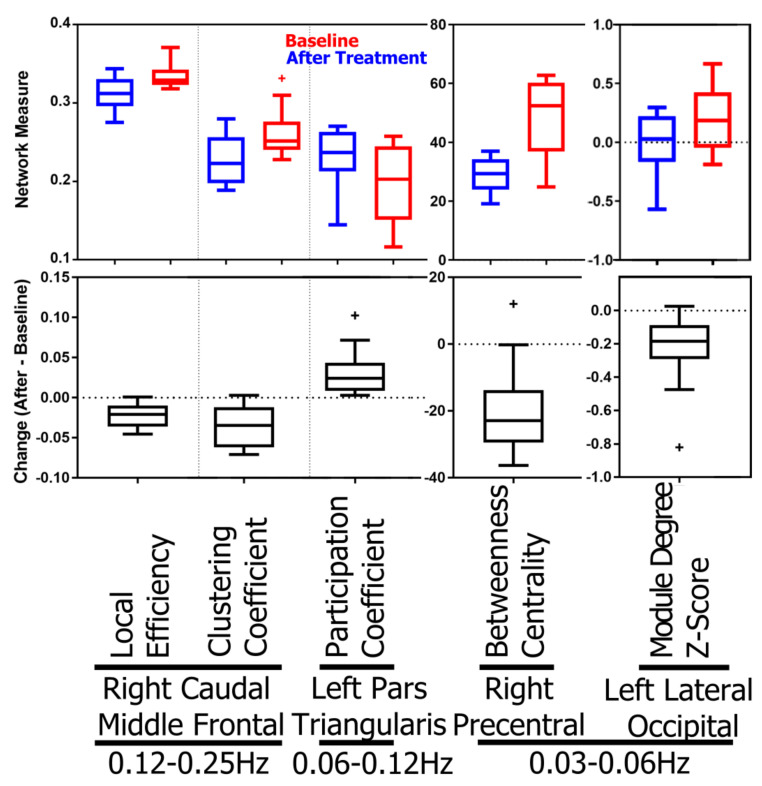
Boxplots for AUCs of topological brain network measures with significant change. The top panel shows the distributions of AUCs before and after the treatment, and the bottom panel shows the distributions of paired AUC changes.

Next, the local network measures associated with each brain region, i.e., network nodes, are analyzed. The results show that treatment-induced topology changes are frequency-specific. After treatment, MDD patients show (*p*-value <0.00066=0.05/76, Bonferroni correction for 76 regions) decreased local efficiency and clustering coefficient in right caudal middle frontal gyrus in the 0.12–0.25 Hz band. In the 0.06–0.12 Hz band, decreased participation coefficient is observed in left pars triangularis. In the 0.03–0.06 Hz band, there are decreases in betweenness centrality in the right precentral gyrus and in within-module-degree z-score in the left lateral occipital cortex. The corresponding *p*-values, effect sizes, and power are listed in Table 3, and the boxplots of the changes and measurements before and after treatment are shown in Figure 3. No significant differences were found in the 0.015–0.03 Hz band. Boxplots in Figure 3 shows a remarkable separation of the betweenness centrality at right precentral in the low-frequency band, 0.03–0.06Hz, and of the local efficiency at right caudal middle frontal gyrus in the high-frequency band, 0.12–0.25 Hz, between baseline and after receiving the SSRI treatment.

The local efficiency of a brain region is characterized by efficiency of paths from it to other regions, and efficiency of a path is defined by the reciprocal of its length [74]. The nodal clustering coefficient measures the possibility that any two neighbors of the node are also connected. The clustering coefficient is also a measure of functional segregation, which is the ability for specialized processing to occur within densely interconnected groups of brain regions [29]. Reduction in both local efficiency and clustering coefficient at right caudal middle frontal gyrus indicates connectivity inhibition. Participation coefficient and betweenness centrality quantify the hubness of a brain region where the participation coefficient characterizes the diversity of intermodular connections of a node, and betweenness centrality is the fraction (shortest paths passing through the node of interest) of all shortest paths in the network. The raised participation coefficient implies the increase in functional integration of left pars triangularis to the rest of the network. Reduced betweenness centrality at the right precentral gyrus shows dwindling of functional integrity. Within-module degree z-score is a localized, within-module version of degree centrality for regions in a brain network heuristically classified into distinct functional groups. Decreased within-module-degree at the left lateral occipital cortex implies reduced connectivity to other regions in the same functional group.

Based on the finding of local efficiency and clustering coefficient change of right caudal middle frontal gyrus in the 0.12–0.25 Hz network, other regions in the same network are also investigated with a lower criteria (*p*-value ≤ 0.05). Results, summarized in Table 4, show both decreased clustering coefficient and decreased local efficiency in bilateral caudal middle frontal gyrus, rostral middle frontal gyrus, superior temporal gyrus, left pars triangularis, putamen, and right superior frontal gyrus. No region is found with either increased clustering coefficient or local efficiency.

**Figure 4:**
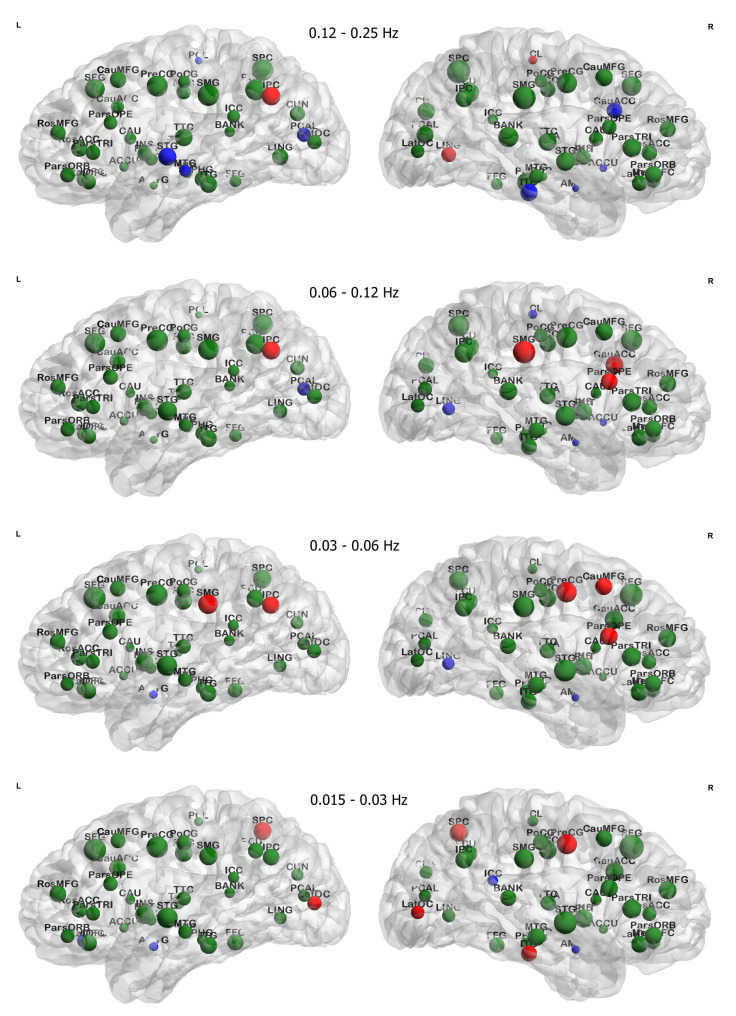
Treatment-induced changes in nodal degree for brain regions are presented. In the figure, before-treatment nodal degree is represented in node size. Significance and direction of change are presented in node color: red nodes for brain regions with (uncorrected *p*-value < 0.05) increased nodal degree after treatment, blue nodes for regions with decreased degree, and green nodes for regions without changes. Statistics for changes of red and blue regions are summarized in Table 5 and full name of brain regions are listed in Table 2.

Figure 4 shows the distribution of nodal centrality, degree, of brain regions in all four networks corresponding to the four frequency bands. Node size represents the baseline degree, and node color indicates the direction (increase/decrease) of changes (uncorrected *p*-value < 0.05) after treatment. The *p*-values, effect size, and power of the altered regions are listed in Table 5. Results show the decreased degree spread over high-frequency to low-frequency bands in regions: bilateral supramarginal gyrus, bilateral lateral occipital cortex, bilateral superior parietal cortex, left inferior parietal cortex, left superior temporal gyrus, right caudal anterior cingulate cortex, right pars opercularis, right caudal middle frontal gyrus, right precentral gyrus, and right inferior temporal gyrus. Changes in the right hemisphere distribute in anterior cortical regions for the high-frequency (0.03 to 0.25 Hz) bands, and posterior cortical regions in the low-frequency (0.015 to 0.03 Hz) band. Regions found to have increased degree are the bilateral amygdala, bilateral paracentral lobule, left pericalcarine cortex, left middle temporal gyrus, left medial orbital frontal cortex, right lingual gyrus, right accumbens, and right isthmus-cingulate cortex. These regions are all associated with emotion regulation, as reported in the previous studies.

#### 3.1.3. Clinical Correlations

**Figure 5:**
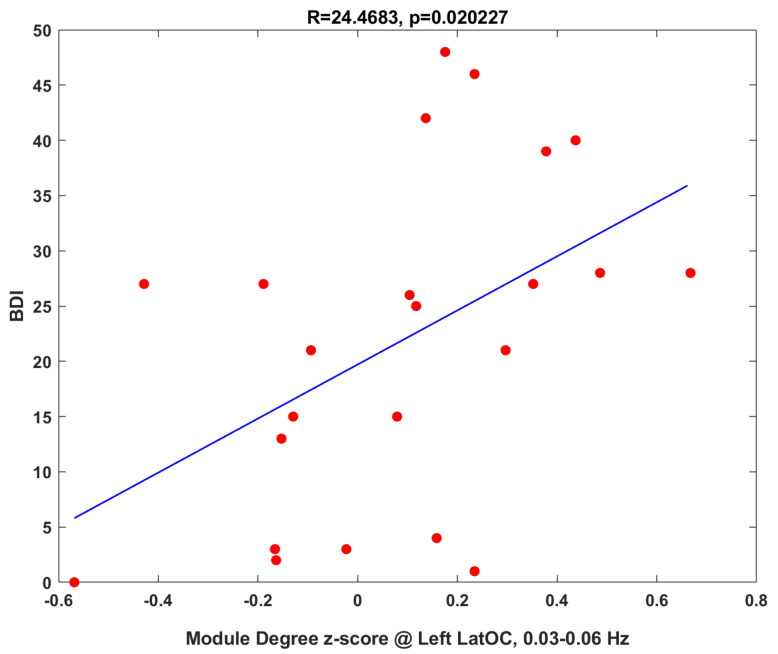
Network measure, module degree z-score of left lateral occipital cortex in the 0.03–0.06 Hz band, correlates with clinical BDI score for both baseline and after treatment with coefficient R=24.4683 and its *p*-value =0.020227. Note that the dependence between baseline and post-treatment measurements could cause the inflation of false positive rate. One subject who could not finish the after-treatment clinical assessment is excluded from the regression.

To further understand the functional significance of the significant results listed in Table 3, we conduct correlation analyses with clinical measures. We first investigate if topological measures correlate with BDI scores, which would have suggested relevance to the disease process. The module degree z-score of left lateral occipital cortex in the 0.03–0.06 Hz band show correlation with the BDI scores (both baseline and after treatment) with *p*-value = 0.02, as shown in Figure 5. Second, we test if the pre-treatment topological measures correlate with the BDI scores and pre-post treatment changes in BDI-II scores to assess if these could serve as treatment outcome predictors. However, no significant correlations were found. Finally, we test correlations between changes in topological measurements and pre-post treatment changes in BDI-II scores, which would have suggested this change as a treatment mechanism. However, no significant correlations were found.

### 3.2. Treatment-Related Changes in Functional Connectivity in Four Frequency Bands

**Figure 6:**
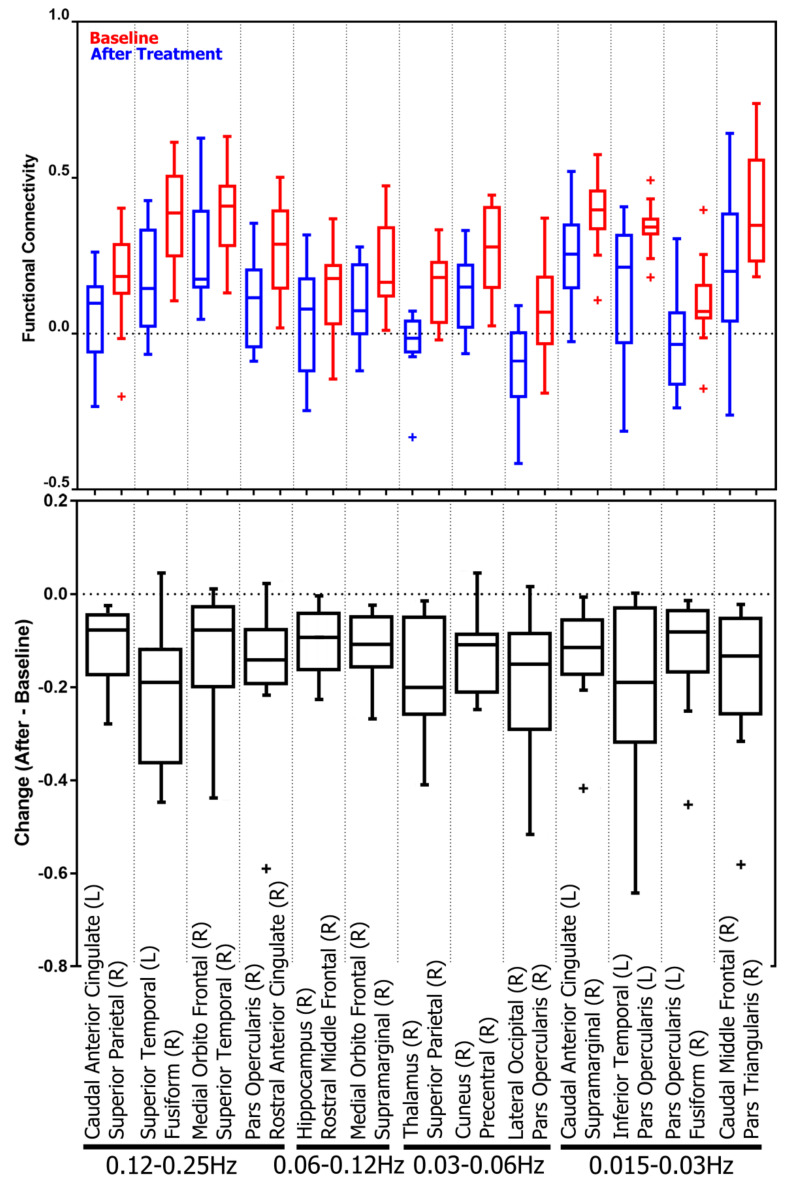
Boxplots for the functional connectivity of the altered connections. Top panel shows the distribution of the functional connectivity before and after the treatment, and the bottom panel shows the distribution of connectivity changes.

The non-parametric sign permutation test identified (*p*-value < 0.05 with Bonferroni correction for 2850 connections) decreased connectivity between right fusiform gyrus and left superior temporal gyrus, between right rostral anterior cingulate cortex and right pars opercularis, between right superior temporal gyrus and right medial orbitofrontal cortex, and between right superior parietal cortex and left caudal anterior cingulate cortex in the 0.12–0.25 Hz network; between right medial orbitofrontal cortex and right supramarginal gyrus and between right hippocampus and right rostral middle frontal gyrus in the 0.06–0.12 Hz network; between right thalamus and right superior parietal cortex, between right cuneus cortex and right precentral gyrus, and between right lateral occipital cortex and right pars opercularis in the 0.03–0.06 Hz network; and between left caudal anterior cingulate cortex and right supramarginal gyrus, between left inferior temporal gyrus and left pars opercularis, between left pars opercularis and right fusiform gyrus, and between right caudal middle frontal gyrus and right pars triangularis in the 0.015–0.03 Hz network. The corresponding *p*-value, effect size, and power are summarized in Table 6, and the boxplots for the distribution of connectivity and its changes are shown in Figure 6. The results show that all the changes involved decreases in connectivity following the treatment. This implies desynchronization of brain activities at the end regions of the listed connections.

While considering loosening the correction to accept *p*-value <0.0005, eight additional connections are included with decreased connectivity after treatment in Table 6. Notably, most of these connections link regions that are known to be involved in emotional processing and regulation, including hippocampus, thalamus, pars opercularis, fusiform, and various cortical regions in the frontal and temporal lobes, respectively. Moreover, the identified connections are mainly part of the ascending and descending serotonergic pathways, which the SSRIs may affect [75].

#### 3.2.1. Subnetwork Analysis

In addition to identifying changes with Bonferroni correction, a false discovery rate (FDR) approach is used as an alternative to identify altered subnetworks. FDR approach controls for a low proportion of false positives in comparison to the Bonferroni correction, which controls the family wise error rate for the probability of making a false positive conclusion.

**Figure 7:**
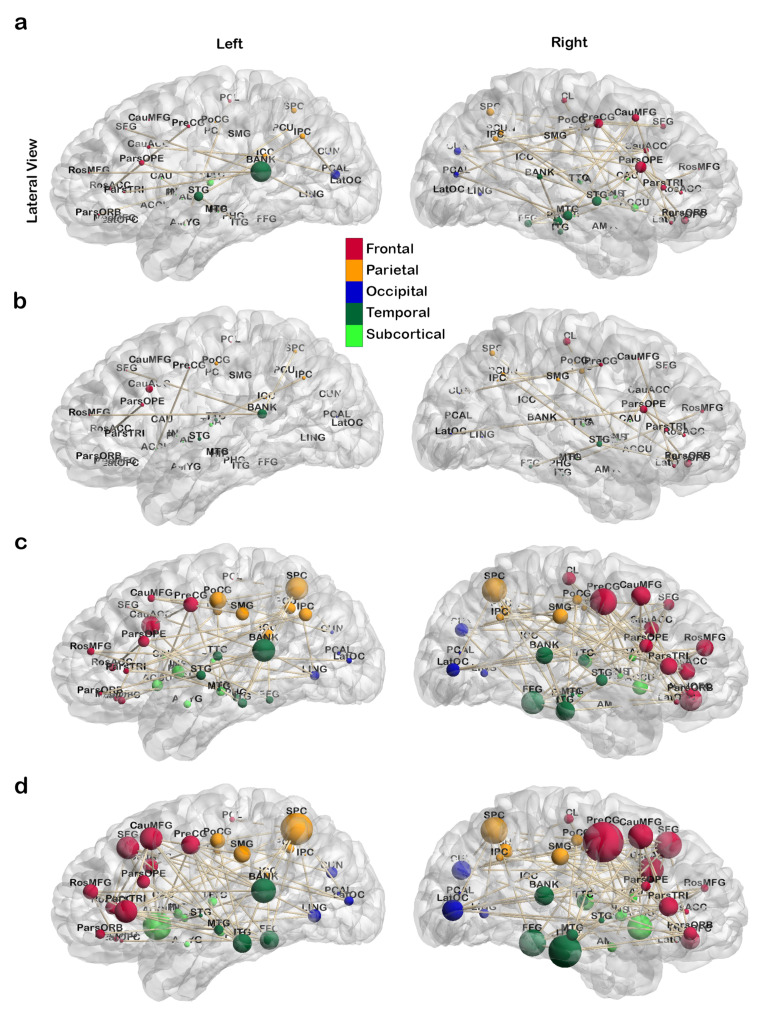
Degree distribution presented in the node size for the significant changes subnetworks identified using Benjamini–Hochberg procedure for FDR control with α=0.05 from the 4 frequency band networks: (**a**) 0.12–0.25 Hz, (**b**) 0.06–0.12 Hz, (**c**) 0.03–0.06 Hz, and (**d**) 0.015–0.03 Hz. Edges are connections associated with significantly (with FDR control α=0.05) changed connectivity.

Figure 7 shows the degree distributions, represented by the size of nodes, for significantly changed subnetworks corresponding to the four frequency bands. The subnetworks are formed by the significantly altered connections determined using the Benjamini–Hochberg procedure for FDR control with α=0.05. There are 80, 42, 210, and 259 connections in the 0.12–0.25 Hz subnetwork, 0.06–0.12 Hz subnetwork, 0.03–0.06 Hz subnetwork, and 0.015–0.03 Hz subnetwork, respectively. Nodes in the subnetworks include regions with significant topological or degree change presented in Section 3.1, such as the amygdala, caudal middle frontal gyrus, pars triangularis, precentral gyrus, and lateral occipital cortex. Many of the frontal regions including bilateral precentral gyrus, bilateral caudal middle frontal gyrus, bilateral caudal anterior cingulate cortex, bilateral pars opercularis, bilateral rostral middle frontal gyrus, bilateral pars orbitalis, and bilateral lateral orbital frontal cortex are shown associated with numerous significant connectivity changes in the two low-frequency bands. In the two high-frequency bands, changes associated with these regions are more significant in the right hemisphere than in the left hemisphere. Left bank superior temporal sulcus shows significant change across all four frequency bands, and bilateral changes of the superior parietal cortex can be found in the two low-frequency bands. Moreover, the degree of bilateral accumbens increases as the frequency decreases. Additionally, Figure 7b shows minimal changes, Figure 7a shows some minor impacted (medium node size) regions in frontal and temporal areas in the right hemisphere, and they become most significant with the presence of parietal regions when frequency decreases as shown in Figure 7c,d. In contrast, the degree (node size) of regions in the right temporal lobe increases when the frequency decreases.

#### 3.2.2. Clinical Correlation: Baseline Connectivity Predicts Treatment Changes

**Figure 8:**
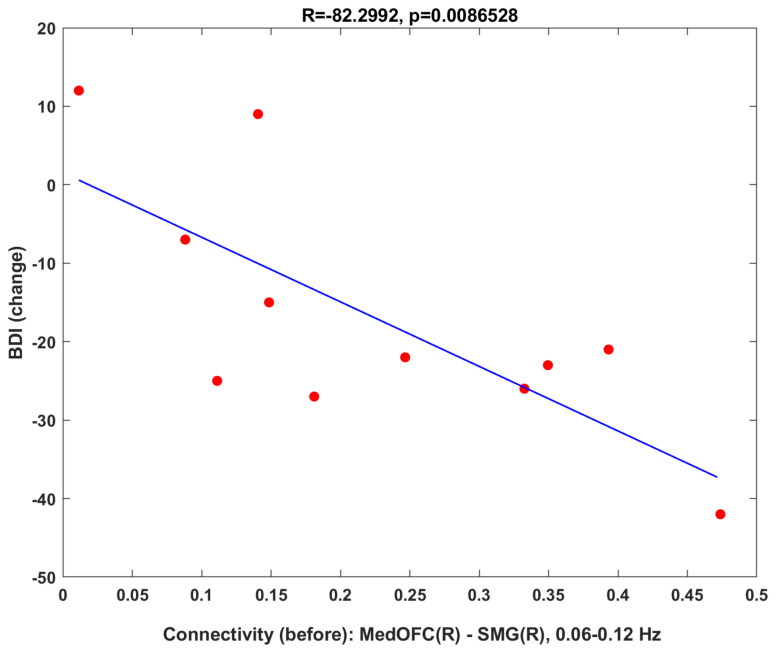
Scatter plot shows correlation between improvement in BDI score and baseline functional connectivity on connection between right medial orbitalfrontal cortex and right supramarginal gyrus in the 0.06–0.12 Hz band (coefficient R=−82.2992 and its *p*-value =0.0086528). Age, gender, and IQ were partialed out. One subject who could not finish the after-treatment clinical assessment is excluded from the regression.

In Figure 8, the change in clinical assessment (BDI score) is to found correlate with the baseline functional connectivity between right medial orbitalfrontal cortex and right supramarginal gyrus in the 0.06–0.12 Hz band. In other words, higher connectivity in this circuit at baseline was associated with greater clinical improvement.

## 4. Discussion

### 4.1. Treatment Impact on Network Topology and Functional Connectivity

This study investigates SSRI-induced changes in brain resting-state functional network topology and connectivity using graph-theory-based network analysis. As hypothesized, patients showed changes after treatment in both topological structures and functional connectivity of brain networks. Additionally, the finding that before-treatment connectivity between right medial orbital frontal cortex and right supramarginal gyrus was associated with greater clinical improvement may suggest a promising avenue for research investigating brain predictors of treatment response that could eventually lead to new clinical tools to help clinicians prescribe the most effective medicine for individual patients.

The graph-theory-based network analysis offers a mathematical schematic for characterizing and quantifying the global and regional topological structure of a brain network. To the best of our knowledge, no prior work has investigated treatment-related changes in brain network structure depending on frequencies for MDD. The most relevant work we can find is regarding the disruption of brain network regardless of frequency [46,76,77,78,79]. Furthermore, to our best knowledge, no prior work has investigated SSRIs-induced topological change in the brain network.

#### 4.1.1. Global Topology

Changes in the global measure of normalized clustering coefficient and smallworldness after treatment are identified. Increased clustering coefficient and smallworldness imply the construction of cliquishness in the network structure. Previous studies have reported decreased clustering coefficient and decreased smallworldness in the MDD patients versus healthy control, which indicates that the treatment change is in the correct direction for improvement [80,81].

#### 4.1.2. Regional Changes and Correlation to Literature

Although thechanges in global measures in Figure 2 are not statistically significant, the changes in local topology support the observed global alterations. These local changes have not yet been reported in any prior SSRI-related study. Therefore, in this section, the changes will be related to previous findings in MDD (may or may not specifically for adolescent).

The reduction in the local efficiency and clustering coefficient at the right caudal middle frontal gyrus in the 0.12–0.25 Hz band is the most significant according to the *p*-value and effect size in Table 3. The finding is new and, to our best knowledge, no literature has reported MDD-related change in this region for adolescents. The change of the betweenness centrality at the right precentral gyrus in the 0.03–0.06 Hz band is the largest according to the effect size in the table. In the region, in MDD patients, previous studies have reported decreased activity, reduced task-related activation, and increased functional connectivity [82,83,84]. For left pars triangularis, previous studies have reported decreased cortical thickness in depression patients, diminishing pars triangularis in functional networks for depression patients, functional connectivity between pars triangularis and frontal eye field as predictors of acute depression treatment outcome, and pars triangularis as a discriminating biomarker between depression patients and healthy controls [85,86,87,88]. For left lateral occipital cortex, in MDD patients, previous studies have revealed abnormal local intrinsic gray-matter connectivity, decreased baseline blood-oxygen level dependent (BOLD) signal, the correlation between suicidality and connectivity between lateral occipital cortex and fusiform, elevated functional activation, and increased cortical surface [89,90,91,92,93,94]. Even though the previous study has discovered similar correlations in MDD patients and correlation between gray matter thickness in areas of parietal and temporal cortices and antidepressant treatments [95], no finding regarding the treatment-related functional changes for regions in Table 3 has been reported before.

Local efficiency is related to the clustering coefficient [96]. The decrease in local efficiency and clustering coefficient after treatment at the caudal middle frontal gyrus suggests the dwindling of the local cliquishness. Table 4 lists brain regions associated with clustering coefficient and local efficiency changes after treatment for uncorrected *p*-value < 0.05 in the 0.12–0.25 Hz network. These regions include bilateral caudal middle frontal gyrus, bilateral superior temporal gyrus, bilateral rostral middle frontal gyrus, left pars triangularis, left putamen, and right superior frontal gyrus. They have been frequently identified as regions correlating with structural and functional abnormalities in depression [84,85,87,88,89,92,97,98,99,100,101,102,103,104,105,106].

In addition to network efficiency and clustering coefficient, we also investigated the centrality of brain regions using the nodal degree, shown in Table 5, which characterizes the importance of a node in the whole brain network. After treatment, decreased centrality is found at the left inferior parietal cortex, right caudal anterior cingulate cortex, left superior temporal gyrus, bilateral supramarginal gyrus, right pars opercularis, right caudal middle frontal gyrus, right precentral gyrus, bilateral lateral occipital cortex, bilateral superior parietal cortex, and right inferior temporal gyrus. Increased centrality is found at the bilateral amygdala, right lingual gyrus, bilateral paracentral lobule, right accumbens, left pericalcarine cortex, left middle temporal gyrus, right isthmus-cingulate cortex, and left medial orbital frontal cortex. Notably, degree changes are frequency-dependent that some regions tend to have changes in the high-frequency band and the other changes tend to be in the low-frequency band, except for the right amygdala, which has changes across all the frequency bands. Brain regions that show altered clustering coefficient, local efficiency, and nodal degree after treatment include the left amygdala, left medial orbitofrontal cortex, and left paracentral lobule.

In the previous cortical thickness study using the same dataset, the increased thickness of the left medial orbitofrontal cortex and the correlations between clinical improvement and increased cortical thickness of the left lateral orbitofrontal cortex and superior frontal cortex were found [107], which potentially relate to increased connectivity. The bilateral middle temporal gyrus also shows a decreased clustering coefficient and local efficiency but not the nodal centrality. All the regions with the topological change due to the antidepressant are in the frontal-limbic system and the default mode network (DMN), which have been previously implicated in MDD [16,46,108,109,110,111].

The amygdala is known to play a large part in processing negative emotions, such as the initiation of fear and stress responses [112,113], and is centrally implicated in MDD [21,114,115,116]. In the amygdala, previous work has reported the volume increase in some studies for patients with depression compared to controls [117], increased BOLD response in unipolar depression [103], and reduced activation after treatment to masked fearful faces [118]. The decreased clustering coefficient, local efficiency, and nodal centrality in the amygdala may reflect an improvement in neurocircuitry. Our previous study using the same dataset but a different approach also discovered changes of resting-state amygdala connectivity, including increased connectivity with the right frontal cortex, right central opercular cortex, and Heschl’s gyrus and decreased connectivity with right precuneus, right posterior cingulate cortex, left supplementary motor area and with right precentral gyrus [21,22]. The temporal lobe is implicated in the regulation of emotional states [119,120] and abnormalities in the temporal cortex have been reported in major depressive disorder in several studies [120,121,122,123,124]. For instance, fMRI studies show increased response in the middle temporal gyrus in MDD patients using a paradigm of evoking effect with picture–caption pairs [123] and using negative emotional scripts [119]. The finding in this study shows decreased cliquishness in the clustering coefficient and local efficiency at the middle temporal gyrus in resting-state functional brain networks after treatment.

#### 4.1.3. Frequency Dependency in Regional Changes

From Table 3 and Table 4 and Figure 4, locations of altered regions show a trend in frequency: Changes in high-frequency bands (0.12–0.25 Hz and 0.06–0.25 Hz) are mainly located in the frontal and temporal areas, and changes in low-frequency bands (0.015–0.03 Hz and 0.03–0.06 Hz) spread into the parietal and occipital areas. This phenomenon is more obvious in the result of FDR subnetworks showing in Figure 7.

In the FDR subnetworks, regions commonly affected across frequency bands are left banks superior temporal sulcus, right pars opercularis, right precentral gyrus, right pars orbitalis, right lateral orbital frontal cortex, bilateral paracentral lobule, and bilateral superior parietal cortex. Changes at regions including bilateral superior temporal gyrus, fusiform gyrus, inferior temporal gyrus, accumbens, supramarginal gyrus, pars triangularis, rostral middle frontal gyrus, superior frontal gyrus, cuneus cortex, pericalcarine cortex, lateral occipital cortex, and right banks superior temporal sulcus appear to be frequency-dependent. In the high-frequency bands, i.e., 0.06–0.12 Hz and 0.12–0.25 Hz, the affected regions are mostly frontal and temporal areas. In the low-frequency bands, i.e., 0.03–0.06 Hz and 0.06–0.12 Hz, parietal and subcortical regions appear affected in addition to the areas identified in the high-frequency networks.

Previous studies have reported subsets of frequency bands as follows: Frequencies between 0.010 and 0.027 Hz may reflect cortical neuronal activity, frequencies between 0.027 and 0.073 Hz may reflect basal ganglia activity, and frequencies between 0.073 and 0.198 Hz and 0.198 and 0.250 Hz have been associated with physiologic noise and white matter signal, respectively [125,126,127,128,129]. Even though there was a debate about whether resting-state fMRI can actually detect white matter activity [130], recent studies tend to support that white matter activity is detectable with resting-state fMRI [131,132].

The frontal lobe is one of the regions that is most consistently identified as associated with MDD [97,133]. Changes in the frontal cortex across all frequency bands may reflect anatomical changes related to white matter. Altered temporal, parietal, and occipital regions in the 0.015–0.03 Hz band are not only primarily related to cortical activities in sensory-motor processing but also participate in a series of higher cognitive functions. The altered network topology in these regions may correspond to potential neural mechanisms mediating the imbalance of these regions’ related functional networks.

#### 4.1.4. Functional Connectivity

In order to expand on our past work where we had previously investigated treatment-related changes in resting-state functional connectivity between amygdala and all other voxels in the brain [22], we also examined functional connectivity between brain regions in the networks at a global level. With Bonferroni correction, thirteen connections are identified. All the connections in Table 6 (*p*-value to <0.0005) show reduced connectivity, and these connections are mostly between limbic, frontal, and temporal regions, as well as the superior parietal cortex.

Moreover, a significant correlation, shown in Figure 8, was found between improvement in clinical assessment and before treatment connectivity of the connection between right medial orbitofrontal cortex and right supramarginal gyrus in the 0.06–0.12 Hz band. Although these correlation analyses are exploratory and the results are not corrected for multiple comparisons, the preliminary findings suggest that the baseline neurological assessment may be used to predict treatment results. MDD is very heterogeneous in that treatment plans have to be individually customized in order to achieve the best result. By better characterizing the neural underpinnings of facts affecting treatment outcomes, this research may pave the way for designing and optimizing treatment for each individual. Taken together, these findings suggest that the network measures derived from graph theory in this study are clinically meaningful, and they may shed light on the neurological underpinnings for better treatment results.

Lastly, we would like to emphasize that this work should be considered as a first and important exploratory study. Treatment-impacted regions, connections, and networks identified in this work have overlap with those implicated in MDD [134]. However, the findings of the SSRI-induced changes and relationships with the clinical assessment reported in this paper require replication with larger data sets. Once validated, these findings could serve as bases for longitudinal studies to answer important questions, such as the following: (1) how network topology and functional connectivity evolve with different lengths of the SSRI intervention; (2) how brain network status correlates with the clinical outcome; (3) how the initial brain network status relates with the intervention outcome; and (4) what and how factors (frequency, strength, etc.) of the intervention contribute to the brain change and clinical improvement.

### 4.2. Methodological Considerations

For the generation of brain functional network and calculation of the network measures, thresholding is utilized to remove insignificant and weak connections that potentially are spurious and could obscure the structure of the significant and strong portion of the network [29]. The threshold is determined per subject in order to ensure the consistent number of connections across subjects for each network density. Any connectivity with a negative correlation coefficient is not considered during the topological analysis, as suggested in the previous study [29].

In this paper, the topological measure-versus-density curves are compared based on the area under curve to test statistical significance, which averages the measure and serves as a scalar summary of the curve values across densities. However, averaging could reduce statistical significance. Another approach is by performing massive comparisons at every single density, which will have higher significance but is also prone to the high type-I error and high sensitivity to noise. To produce a better comprehensive examination of the entire topological structure of the original weighted connectivity graph, in the future study, AUCs for subdivided density ranges or AUCs calculated based on weighted averaging will be investigated to achieve balance between significance and the control of type-I error.

In a connectomic study, type-I error control is necessary for multiple comparisons in finding the changes from 2850 connections in a brain network. A previous study has suggested that, in a connectomic analysis, ruling out unnecessary candidates for comparison using existing knowledge could result in a smaller type-I error and an improved significance [72]. Therefore, in this study, we employed the traditional false-discovery rate (FDR) control procedure to create a *significantly impacted subnetwork*. Unlike the more restricted family wise error rate control procedure, the FDR procedure ensures the enclosure of all the true positives and offers a guarantee on the ratio of false-positive to the true positive [135]. By excluding the irrelevant part of the network, the preliminary result shows the significantly affected connections between cortical and subcortical regions as part of the ascending and descending pathways that are expected to respond to SSRIs and highlights the subcortical regions associated to these connections. Future investigation will result in a complete study of the topological structure of the subnetwork.

The parcellation of the brain directly determines the size of the brain network and the number of fMRI signals being averaged while computing the seed-based functional connectivity, i.e., Pearson correlation between the average (representative) signal between a pair of regions. In this paper, the commonly used Desikan–Killiany atlas (82 cortical and subcortical regions) is used to define nodes in the functional brain networks. The regions are relatively large compared to the imaging resolution, which means that the advantage of current advanced imaging technology is not fully utilized. Additionally, brain networks derived using different parcellation schemes may show different topological structures. Therefore, it will be interesting to perform similar studies using various atlases and to compare the results.

Additionally, here, functional connectivity is defined based on Pearson correlation coefficients that only characterizes the linear relationship between time series. Some other types of connectivity measures, such as coherence and mutual information, could be utilized to account for a more complicated relationship, such as timing-lag and predictability, between two time series.

Lastly, recent fMRI studies have reported that resting-state functional brain connectivity is highly dynamic [136,137]. In this study, the brain network is formulated as a static network by considering an average representation for the entire period during the resting-state fMRI acquisition, which is consistent with many of the current functional connectomic studies [37,44,46]. However, investigation of the dynamical changes in functional connectivity and network topology is necessary for future work.

#### Limitations

The small sample size, N=12, is the major constraint for this study. Although the statistical power for functional connectivity is acceptable, 0.2968–0.9822 (mean 0.6811, SD 0.1838) in Table 6, the power is very limited for global network measures, 0.2834–0.9962 (mean 0.6820, SD 0.1804) in Table 3 and Table 4, and for local measures, 0.2110–0.7240 (mean 0.4443, SD 0.1272) in Table 5. Insufficient power would downgrade the ability of discovering a true effect and increase the chance that a significant result does not imply a true effect [138].

In addition to the small sample size, the lack of placebo control that was scanned at similar intervals prevents us from concluding that the changes observed in neural networks are all necessarily due to the treatment, as opposed to spontaneous changes that would have happened anyway. Therefore, the result should be considered preliminary and needs to be validated on large samples in future works.

The absolute values of effect size (Cohen’s d) for the five topology measures listed in Table 3 range from 0.7271 to 1.8910 (mean 1.1646, SD 0.4036), indicating large to very large effects [68,139]. Those for connectivity changes that listed in Table 6 range from 0.5818 to 1.6582 (mean 1.0389, SD 0.2697), which indicate medium to very large effects [68,139]. These effect sizes are comparable to the previously reported effect sizes in 26,841 statistical records from 3801 cognitive neuroscience and psychology papers [140]. With the large power for results in Table 3 and Table 6, we can eliminate the concern that an effect size could be overestimated by low statistical power [138]. Therefore, even though preliminary findings in this study are promising and could be significant after suitable validation on a large dataset.

## 5. Conclusions

In this paper, graph-theory-based complex network analysis has been applied to investigate SSRI-induced alterations of topological organizations and connectivity in resting-state functional brain networks. In the 0.12–0.25 Hz functional brain network, after treatment, MDD patients show decreased local cliquishness and nodal centrality in the middle frontal and superior temporal regions. Furthermore, subnetworks with significantly altered connections present frequency-dependent changes in functional connectivity. Additionally, the baseline connectivity of connection between the right medial orbitofrontal cortex and right supramarginal gyrus in the 0.06–0.12 Hz band is found to be correlated with clinical improvement, which may signal its potential usefulness in predicting treatment outcome, pending confirmation with future studies. The findings of this work may help in gaining new knowledge into the neural underpinnings of the SSRI treatment effect. However, due to the limitation of small sample sizes and lack of placebo control, the results should be viewed as exploratory and need to be validated on large samples in future works. Future efforts will be directed towards studying functional brain networks constructed with different node sets and connectivity measures, exploring the dynamic network structure across time, and testing the results on a larger sample size.

## Figures and Tables

**Table 1:** Demographic Information.

	Baseline	Post Treatment
Gender	3 male, 9 female	
Handedness	11 right, 1 left	
IQ (mean ± SD)	104.58±17.19	
Age (years, mean ± SD)	15.68±2.12	15.89±2.12
BDI-II (mean ± SD)	30.33±8.91	12.45±14.14
Duration of illness ${}^{*}$ (years, mean ± SD)	4.1±2.58	

SD: standard deviation; BDI: Beck Depression Inventory II [50]; * from the onset of the first episode, which may not be the current episode.

**Table 2:** List of regions-of-interest (ROIs) from Desikan atlas.

No.	Region of Interest	Abbr.	No.	Region of Interest	Abbr.	No.	Region of Interest	Abbr.
1	Banks superior temporal sulcus	BANK	14	Parahippocampal gyrus	PHG	27	Superior parietal cortex	SPC
2	Caudal anterior cingulate cortex	CauACC	15	Paracentral lobule	PCL	28	Superior temporal gyrus	STG
3	Caudal middle frontal gyrus	CauMFG	16	Pars opercularis	ParsOPE	29	Supramarginal gyrus	SMG
4	Cuneus cortex	CUN	17	Pars orbitalis	ParsORB	30	Transverse temporal cortex	TTC
5	Fusiform gyrus	FFG	18	Pars triangularis	ParsTRI	31	Insula	INS
6	Inferior parietal cortex	IPC	19	Pericalcarine cortex	PCAL	32	Thalamus	THA
7	Inferior temporal gyrus	ITG	20	Postcentral gyrus	PoCG	33	Caudate	CAU
8	Isthmus-cingulate cortex	ICC	21	Posterior cingulate cortex	PCC	34	Putamen	PUT
9	Lateral occipital cortex	LatOC	22	Precentral gyrus	PreCG	35	Pallidum	PAL
10	Lateral orbitalfrontal cortex	LatOFC	23	Precuneus gyrus	PCUN	36	Hippocampus	HIP
11	Lingual gyrus	LING	24	Rostral anterior cingulate cortex	RosACC	37	Amygdala	AMYG
12	Medial orbitalfrontal cortex	MedOFC	25	Rostral middle frontal gyrus	RosMFG	38	Accumbens	ACCU
13	Middle temporal gyrus	MTG	26	Superior frontal gyrus	SFG			

**Table 3:** Brain regions and altered topological measures (*p*-value < 0.05 with Bonferroni correction for 76 regions).

Frequency Band	Brain Region	Network Measure	Uncorrected *p*-Value	Effect Size	Power
0.12–0.25 Hz	Right caudal middle frontal gyrus	Local Efficiency	0	−1.2021	0.8376
		Clustering Coefficient	0	−1.1443	0.8004
0.06–0.12 Hz	Left pars triangularis	Participation Coefficient	0	0.7271	0.4291
0.03–0.06 Hz	Right precentral gyrus	Betweenness Centrality	0.000244	−1.8910	0.9962
	Left lateral occipital cortex	Within-Module-Degree Z-score	0.000244	−0.8587	0.5571
0.015–0.03 Hz	–None–				

**Table 4:** Brain regions showing (uncorrected *p*-value < 0.05) altered clustering coefficient and local efficiency in the 0.12–0.25 Hz network.

Brain Region	Clustering Coefficient			Local Efficiency		
	*p*-Value	Effect Size	Power	*p*-Value	Effect Size	Power
Right caudal middle frontal gyrus	0	−1.1443	0.8004	0	−1.2021	0.8376
Left caudal middle frontal gyrus	0.0371	−0.6342	0.3424	0.0247	−0.5485	0.2693
Right superior temporal gyrus	0.0037	−1.3222	0.8995	0.0081	−1.2372	0.8578
Left superior temporal gyrus	0.0161	−0.9318	0.6264	0.0105	−1.1316	0.7916
Right rostral middle frontal gyrus	0.0369	−0.7923	0.4924	0.0427	−0.8007	0.5006
Left rostral middle frontal gyrus	0.0271	−0.8621	0.5603	0.0437	−0.8181	0.5175
Left pars triangularis	0.0139	−0.7683	0.4690	0.0195	−0.7555	0.4565
Left Putamen	0.0269	−0.6604	0.3662	0.0078	−0.7819	0.4823
Right superior frontal gyrus	0.0427	−0.6444	0.3517	0.0151	−0.7948	0.4948

**Table 5:** Effect size, *p*-value, and power of brain regions that show changes in degree after treatment in at least one of the frequency bands. (Shadowed values indicate uncorrected *p*-value < 0.05). Sign and magnitude of the effect size indicate the direction and significance of changes.

	0.12–0.25 Hz			0.06–0.12 Hz			0.03–0.06 Hz			0.015–0.03 Hz		
Brain Region ${}^{*}$	*p*-Value	Effect Size	Power	*p*-Value	Effect Size	Power	*p*-Value	Effect Size	Power	*p*-Value	Effect Size	Power
Right AMYG	0.0229	0.9426	0.6364	0.0251	0.9588	0.6513	0.0471	0.8203	0.5197	0.0310	0.8350	0.5340
Right LING	0.0396	0.6730	0.3779	0.0093	0.9946	0.6830	0.0439	0.6990	0.4022	0.2832	0.2845	0.1072
Left IPC	0.0134	−0.7909	0.4910	0.0095	−0.8019	0.5017	0.0493	−0.6442	0.3514	0.1904	−0.3872	0.1577
Right CauACC	0.0195	−0.5995	0.3119	0.0061	−0.7787	0.4791	0.0750	−0.5659	0.2834	0.0764	−0.5485	0.2693
Right PCL	0.0134	1.0429	0.7240	0.0376	0.8733	0.5711	0.0732	0.5933	0.3066	0.1189	0.3374	0.1311
Right ACCU	0.0117	0.7705	0.4711	0.0364	0.4711	0.2110	0.2185	0.1645	0.0688	0.4985	0.0057	0.0500
Left PCAL	0.0098	0.6922	0.3958	0.0483	0.5589	0.2777	0.0942	0.5722	0.2887	0.1550	0.4514	0.1976
Left MTG	0.0435	0.7227	0.4249	0.0598	0.6256	0.3347	0.0962	0.5306	0.2551	0.1958	0.3376	0.1312
Left PCL	0.0273	0.8818	0.5792	0.0701	0.6791	0.3835	0.1899	0.3893	0.1589	0.2307	0.2698	0.1013
Left STG	0.0305	−0.5878	0.3018	0.0959	−0.4065	0.1690	0.4817	−0.0101	0.0501	0.0911	−0.3201	0.1228
Right SMG	0.2937	−0.1640	0.0687	0.0376	−0.5360	0.2594	0.0562	−0.5787	0.2941	0.1162	−0.4814	0.2183
Right ParsOPE	0.0581	−0.4317	0.1846	0.0259	−0.8706	0.5685	0.0474	−0.8409	0.5398	0.2766	−0.2684	0.1008
Right CauMFG	0.1460	−0.4486	0.1957	0.0713	−0.6097	0.3207	0.0154	−0.8278	0.5270	0.0735	−0.5341	0.2578
Left SMG	0.2910	−0.1788	0.0722	0.0596	−0.3989	0.1645	0.0439	−0.5430	0.2649	0.0854	−0.4743	0.2133
Right PreCG	0.0981	−0.5977	0.3103	0.0935	−0.5981	0.3107	0.0488	−0.7695	0.4702	0.0364	−0.8346	0.5336
Left AMYG	0.1047	0.4765	0.2149	0.0735	0.4982	0.2305	0.0425	0.5281	0.2531	0.0288	0.6769	0.3815
Right ICC	0.2344	0.2565	0.0963	0.1973	0.3520	0.1385	0.1230	0.4846	0.2206	0.0374	0.7931	0.4932
Right LatOC	0.4194	0.0826	0.0547	0.4849	0.0191	0.0503	0.2109	−0.2854	0.1076	0.0144	−0.6771	0.3817
Right SPC	0.1628	−0.4106	0.1715	0.0867	−0.4923	0.2262	0.0715	−0.5109	0.2399	0.0317	−0.6434	0.3507
Left LatOC	0.3459	−0.1780	0.0721	0.3745	−0.1399	0.0636	0.2603	−0.2623	0.0985	0.0188	−0.6102	0.3212
Left MedOFC	0.2705	0.1984	0.0775	0.1609	0.3556	0.1404	0.1006	0.3966	0.1632	0.0330	0.6721	0.3770
Left SPC	0.4883	0.0102	0.0501	0.3472	−0.1433	0.0642	0.1157	−0.4680	0.2089	0.0186	−0.7846	0.4849
Right ITG	0.0442	−0.5813	0.2963	−0.0867	0.3706	0.1484	−0.0696	0.4827	0.2193	0.0061	−0.7844	0.4847

* Abbreviations defined in Table 2.

**Table 6:** Functional connections having connectivity change with *p*-value less than 0.0005.

Frequency Band	Connection		Uncorrected *p*-Value	Effect Size	Power
	Region 1	Region 2			
0.12–0.25 Hz	Right fusiform gyrus	Left superior temporal gyrus	0 ${}^{1}$	−1.3560	0.9133
	Right rostral anterior cingulate cortex	Right pars opercularis	0 ${}^{1}$	−1.0312	0.7143
	Right superior temporal gyrus	Right medial orbitofrontal cortex	0 ${}^{1}$	−0.8439	0.5427
	Right superior parietal cortex	Left caudal anterior cingulate cortex	0 ${}^{1}$	−0.7088	0.4116
	Right inferior temporal gyrus	Left banks superior temporal sulcus	0.0002441	−1.0550	0.7338
	Right rostral anterior cingulate cortex	Left pars opercularis	0.0002441	−1.0415	0.7228
0.06–0.12 Hz	Right medial orbitofrontal cortex	Right supramarginal gyrus	0 ${}^{1}$	−0.8198	0.5192
	Right hippocampus	Right rostral middle frontal gyrus	0 ${}^{1}$	−0.5818	0.2968
	Right fusiform gyrus	Left superior temporal gyrus	0.0002441	−1.1454	0.8012
	Right pars opercularis	Right rostral anterior cingulate cortex	0.0002441	−0.9197	0.6151
	Right rostral anterior cingulate cortex	Left pars opercularis	0.0004883	−0.9616	0.6538
	Right precentral gyrus	Left caudal anterior cingulate cortex	0.0004883	−0.8591	0.5575
0.03–0.06 Hz	Right thalamus	Right superior parietal cortex	0 ${}^{1}$	−1.5722	0.9707
	Right cuneus cortex	Right precentral gyrus	0 ${}^{1}$	−0.8869	0.5841
	Right lateral occipital cortex	Right pars opercularis	0 ${}^{1}$	−1.2856	0.8828
	Right thalamus	Left superior parietal cortex	0.0002441	−1.6582	0.9822
0.015–0.03 Hz	Left caudal anterior cingulate cortex	Right supramarginal gyrus	0 ${}^{1}$	−0.9047	0.6010
	Left inferior temporal gyrus	Left pars opercularis	0 ${}^{1}$	−1.2272	0.8522
	Left pars opercularis	Right fusiform gyrus	0 ${}^{1}$	−0.8613	0.5595
	Right caudal middle frontal gyrus	Right pars triangularis	0 ${}^{1}$	−0.8023	0.5021
	Left fusiform gyrus	Right precentral gyrus	0.0002441	−1.2953	0.8874

${}^{1}$ The threshold to be significant with Bonferroni correction for 2850 connections is $1.75\times 10^{-5}$.

## Data Availability

The data presented in this study are available on a limited basis on request from the corresponding author. The data are not publicly available due to privacy restrictions.
